# Surgical and Pathological Cases

**Published:** 1831-12

**Authors:** James Laidlaw

**Affiliations:** Member of the Royal Colleges of Surgeons of London and Edinburgh, and formerly House Surgeon to the Middlesex Hospital.


THE LONDON

### Medical and Physical Journal.

394, VOL. LXVI.] DECEMBER 1831. [66, *New Series*.

"For many fortunate discoveries in medicine, and for the detection of numerous errors, the world is indebted to the rapid circulation of Monthly Journals; and there never existed any work to which the Faculty, in Europe and America, were under deeper obligations than to the 'Medical and Physical Journal of London,' now forming a long but invaluable series."—RUSH.

### ORIGINAL PAPERS, AND CASES,

### OBTAINED FROM PUBLIC INSTITUTIONS AND OTHER AUTHENTIC SOURCES.

### SURGICAL AND PATHOLOGICAL CASES.

*Surgical and Pathological Cases.* By JAMES LAIDLAW, Member of the Royal Colleges of Surgeons of London and Edinburgh, and formerly House Surgeon to the Middlesex Hospital.

*Case of Foreign Body in the Œsophagus.* ON the 13th of February, 1830, the relatives of a man, named Thomas Murphy, and who they stated had died the day previous, applied to me, requesting that I would make an examination of the body, as they believed he had died in consequence of a bone sticking in his throat, and they were particularly desirous to ascertain if that had been the case. The history which they gave of the accident was, that, ten days ago, this unfortunate fellow (whom they described as a remarkably fine young man, twenty years of age,) was making his dinner of some mutton broth, and, being pressed for time, he hastily swallowed a quantity of it from the basin, and immediately felt that something was sticking in his throat: he in vain attempted to get it down by swallowing, or to get it up by making himself sick: he easily made himself vomit by tickling the fauces, and thus completely emptied the stomach, but still the substance could not be dislodged, and, after wearying himself with repeated attempts, he went to bed. Having passed a restless night, and finding himself getting worse, he became alarmed, and in the morning applied to a professional gentleman in the neighbourhood, who passed an elastic tube into the œsophagus, but was unable to detect the presence of any foreign substance; and, as the patient vomited freely, he was led to believe that the irritation, and consequent inflammation, remaining from a body having been lodged there, and which he supposed must have been swallowed, was quite sufficient to account for the sensations of the patient: he, therefore, merely gave him some opening medicine, and directed him to call again on the following day. To this the poor fellow unfortunately paid no attention, but, upon going home, he took to bed, telling his friends he had no hopes, he was sure he should die. His distress had by this time greatly increased: his breathing was very laboured, and his countenance was livid and anxious; he obtained no rest, and could not lie down in bed: he expressed no wish to have medical assistance, as he felt convinced it would be unavailing; and having sent for his priest, and made his necessary domestic arrangements, he looked forward to death rather as a relief from his suffering, than as an event to be regarded with apprehension: he thus continued getting daily worse, and on the tenth day he died apparently suffocated.

Upon opening the body, and removing the larynx, pharynx, trachea, and œsophagus, the substance which had been swallowed, and which had produced all the mischief, was found to be one of the vertebræ of a sheep's tail, and was still firmly fixed in the upper part of the œsophagus: it is difficult, indeed, to conceive any thing which would have been so troublesome to remove. One of its processes had ulcerated through the anterior part of the gullet, and had perforated the trachea, into which it projected immediately below the cricoid cartilage; another process had perforated the œsophagus on its posterior surface, and was sticking against the cervical vertebræ; and a third process was firmly fixed in a perforation it had made laterally. The trachea and œsophagus were both in a high state of inflammation, and those portions of them surrounding the perforations made by the processes of the bone had a sloughy appearance.

As the foreign body was so firmly wedged in and entangled, it is evident that any attempts made from the mouth, either to draw it out or push it down into the stomach, must have been completely unsuccessful, and would have only added to the pain and irritation. If the poor fellow had been fortunate enough to have had the assistance of a surgeon, who had been sufficiently bold and skilful to undertake the operation of œsophagotomy, under such very disadvantageous circumstances as the above, the patient might possibly have been saved; but when it is considered that the bone had already perforated the trachea, and was lodged below the cricoid cartilage, and would have required the incision to have been made very low in the neck, and of such a size as would have admitted of the introduction of instruments to break the bone in pieces, inasmuch as it would have been impossible to have disentangled it so as to allow of its being withdrawn entire, I think, when these circumstances are added to the usual difficulties of the operation, there could have been very little hope of a favorable result.

Thus was this fine young fellow destroyed without even an attempt being made to save him; and what appears most inexplicable is, that his relatives, who were in good circumstances, should not have sought medical assistance, even when they saw him dying, but, after his death, applied to me to open him, merely for the purpose of gratifying their curiosity.

*Case of a Needle lodged in the Pharynx.* Wm. Packwood, a tailor, applied to me on the 21st March, 1829, and with great difficulty told me that, as he was sitting at his work some hours previously, he put several needles into his mouth, one of which had slipped into his throat, and was still sticking there. He had made himself sick, hoping by that means to dislodge it, but it only increased his distress, and, becoming greatly alarmed, he immediately sought medical assistance.

Upon looking into his throat, I could not perceive it, (as he stated some of his friends had,) and was inclined to think he must have swallowed it; but, upon thrusting my fingers into the pharynx, I could, with a little difficulty, just touch the needle with the tip of my finger, and feel that it was lying firmly fixed obliquely across the pharynx, one end of it sticking in the root of the tongue immediately above the epiglottis, and the other in the right side of the pharynx, below and behind the arches of the palate. I then passed a pair of long forceps, directed by the fore-finger of the left hand, into the throat, and, having seized the needle firmly with them, I disengaged the end of it, which was sticking in the lateral part of the pharynx, by pressing outwards with the finger, and was thus enabled to turn and withdraw it. He was directed to gargle his throat frequently with tepid water, and in a day or two there was no inconvenience remaining.

In this case it was fortunate for the patient that no attempts were made to pass a probang into the œsophagus, as it could only have been productive of mischief by the laceration it would have caused, independent of the hazard of breaking the needle; for, had the latter happened, in all probability the portion sticking in the root of the tongue would have fallen into the larynx, which would have been unguarded at this moment by the epiglottis, in consequence of the tongue being depressed to allow of the examination of the throat.

The consequences might have been similar had the needle slipped from the grasp of the forceps at the time of its extraction.

*Curious Cases of Pseudo Syphilitic Affection, with subsequent Sympathetic Amaurosis in both the Patients.* Thomas Welsh, twenty-seven years of age, by trade a tailor, applied to me on the 6th of June, 1829, complaining of pains in his bones and soreness of the throat. He stated, upon inquiry, that, about three months back, he had married a woman upon whose virtue he had the greatest reliance, and that very shortly after his marriage he discovered a sore upon his penis: as this gave him little inconvenience, he paid no attention to it, and, in the course of another week or two, he observed a small swelling in the right groin, which was sometimes slightly painful, but not so much so as to cause him to seek medical assistance. Both the sore on the penis and the swelling in the groin remained stationary for a few weeks, and he was then attacked with sore throat, and an eruption broke out upon various parts of the body: to these symptoms succeeded violent pains in his bones, which totally prevented his working, and compelled him to seek the advice of a surgeon. Upon examining him, I found a sore upon the back of the glans penis, completely circular, and about the size of a split pea; the edges were white and indurated, but the sore was more superficial than is generally the case with true chancres. Upon looking into his throat, several small ulcers were apparent, particularly upon the tonsils: his face was covered with a dark-coloured eruption, throwing off branny scales: his general health appeared to be much out of order, and he complained of a good deal of pain in the right hypochondriac region. The pains in the limbs were much increased at night, and prevented his sleeping. The eruption was most apparent upon the face, but the extremities and trunk were also a good deal marked by it.

As the patient positively denied ever having had syphilis in any shape, and as the symptoms were of a dubious nature, although certainly very suspicious, I determined to treat the case for a week or two without mercury, and endeavour to get him into a little better general health: I, therefore, directed him to take a dose of jalap and calomel immediately, so as to produce a copious evacuation from the bowels, and prescribed an alum gargle for the throat. I also desired him to drink daily a pint of the Decoct. Sarsaparillæ co., with fifteen drops of the Acid. Nitric. No local application was directed for the sore on the penis, but he was cautioned to keep it very clean by bathing it frequently in tepid water. This treatment had been continued for a very few days only when an evident amendment took place: the sore upon the penis began to heal rapidly, and the eruptions became gradually paler; the throat was also much improved. At the end of a fortnight, the whole of the symptoms were removed; the cicatrix remaining from the sore on the penis was scarcely perceptible; and he said he had not, for a very long period, been in such good health and spirits.

A few days before I dismissed him, he requested me to see his wife, who, he stated, had an eruption and sore throat similar to that from which he had been relieved, and I found, upon examining her, that she was affected in exactly the same way, though not quite so severely as he had been: she was much out of health; her bowels had not been moved for several days, the tongue was covered with a brown fur, and the breath was very offensive. There were several small ulcers in the throat and upon the tonsils, and the eruption was chiefly confined to the face and arms. She admitted having had, some time after her marriage, a slight discharge from the vagina, but said that it went away in a day or two.

She was directed to take a dose of calomel and rhubarb immediately, and some Infus. Sennæ, with Sulph. Magnes., every two hours, till the bowels were freely evacuated; and as I had been so successful in the husband's case with the sarsaparilla, I ordered her to take it in the same quantity. In a week she became much better, and in a fortnight the sore throat, eruption, and every other symptom, had disappeared.

Of the above parties I saw nothing more for upwards of two months, when one morning the man applied to me on account of an affection of one of his eyes: he stated that, about three weeks back, while looking out of the window early one morning, he first felt a severe pain in his left eye, and shortly afterwards a cloudiness of the vision (as he expressed it) took place; he was surprised at finding, upon closing the other eye, that he could not distinguish objects. As he could still see very well with one eye, he thought it would go off, and merely took some opening medicine, which he said relieved the pain, but the vision was not restored in any degree. He stated that his wife had had a similar attack in one of her eyes, and intended to apply to me the following day, but she had been under the care of a medical gentleman since the attack, who had not been able to relieve her.

Upon examining his eye, I found the conjunctiva slightly inflamed, the pupil was widely dilated, and the iris totally insensible to the stimulus of a powerful light; the iris of the other eye at the same time acting naturally. Upon looking carefully into the diseased eye, I could not detect any other morbid appearances than the above. His general health was much disordered: he complained of pain in the side, loss of appetite, and great depression of spirits; his tongue was covered with a brown fur; and he had had no evacuation from the bowels for more than a week. ℞. Hydrarg. Subm. gr. v., Pulv. Jalap. ℈i. fiat pulv. h. s. s. He was directed to take a senna draught in the morning, if his bowels were not freely opened during the night; a slightly astringent lotion was given him for the eye.

When I saw him the next morning, the medicine had not operated, and I desired him to repeat the draught every two hours, as long as he should find it necessary; prescribed some calomel and rhubarb for him at night; and ordered a blister to be applied to the temple, and kept open.

In the course of a few days his health began to improve, but the eye continued much the same: no alteration was, however, made in the medicine, and a small dose of calomel and rhubarb was given every night, and occasionally an aperient draught in the morning. After continuing the medicine about a fortnight, the eye began to improve, the iris acted slightly, and he in some degree recovered his sight, but complained of the eye being weak: he ultimately, by the above treatment, recovered the perfect use of the eye, and could see as well as with the other.

It has been stated above that the wife had had an attack similar to that of the husband, and, what is very remarkable, she was taken with it suddenly on the same day. The account she gave of it was, that on the evening of the day on which her husband first lost the sight of his eye, she was suddenly seized with a violent pain in one of her eyes; she went to bed immediately, and in the morning found the eye violently inflamed, and the vision totally gone. She became alarmed, and applied to a gentleman celebrated as an oculist, who ordered her some calomel and jalap to be taken directly, and directed twenty leeches to be applied to the temples, and some solution of extract of belladonna to be dropped into the eye occasionally. The following pill was also directed to be taken every night: ℞. Pil. Hydrarg. gr. v.; Opii, gr. ss. fiat pil. This treatment was persevered in for about a fortnight, and then, not finding herself as much better as she expected, she placed herself under my care.

Upon examining the eye, the conjunctiva was observed to be greatly increased in vascularity; the pupil was widely dilated, and the iris, instead of being like that of the other eye, of a bright grey colour, was changed to a dirty brown. The iris did not act upon being exposed to a strong light; she could not see at all when the sound eye was closed. Her health was greatly impaired: the bowels were confined, and the tongue furred; she suffered a good deal of pain from the inflammation of the conjunctiva, and obtained very little rest at night. A smart dose of calomel and jalap was given to her, so as to freely empty the bowels; the conjunctiva was scarified, and leeches and a blister applied to the temple.

In the course of a few days, the inflammation was a good deal subdued, but no alteration had taken place in the state of the iris; she was still totally blind with the diseased eye. I directed her to keep the blister on the temple open with savine ointment, gave her a weak solution of Zinci Sulph. for the eye, and prescribed a pill of calomel and opium to be taken every night.

By continuing this treatment for a fortnight, an evident improvement took place: there was no longer any inflammation of the conjunctiva, the iris acted slightly, and she could imperfectly distinguish objects. The disease then became stationary, and for another week no alteration was apparent, and my patient being relieved from pain, and becoming impatient at not getting well more speedily, I suppose, applied to some other practitioner. I have never been able to learn in what manner the case terminated; but I have no doubt, if the treatment adopted had been persevered in, a complete restoration of the vision might have been effected.

The above cases I have considered worthy of record, inasmuch as they shew an unusual coincidence in the circumstance of the husband and wife being both twice affected with a similar disorder at the same time. That such might occur once, where the complaint was not contagious, is not unlikely, but that they should both be attacked with such a disease as amaurosis on the same day, and in each case it should be confined to one eye, is somewhat extraordinary. It is quite impossible either of them could have been counterfeiting, as, in order to satisfy myself, I held a lighted candle before the diseased eye, without producing any change in the pupil, which was widely dilated, and which afterwards contracted and dilated naturally. It can only be attributed to the very irregular manner in which the parties lived, as the husband admitted that they both frequently ate and drank to excess.

*Wound of the Eye, with Loss of a Portion of the Iris.*

James Funge applied at the Middlesex Hospital, in consequence of having received a severe wound of the right eye, by the bursting of a soda-water bottle, one of the fragments of which had flown into the eye. Upon examining it, it was found that the upper and inner portion of the cornea was divided, and the sclerotic cut to the extent of about three lines; the anterior chamber was filled with florid blood. Leeches were plentifully applied to the temple, and the eye was kept closed, lead lotion being applied externally; and a smart dose of opening medicine was given him.

A slight degree of inflammation took place, but was quickly subdued, and in a few days the wound was quite healed, and the blood in the anterior chamber absorbed. It was then discovered that the iris had been wounded, and about a third of it destroyed; but this was no hindrance to vision, as he could read as well with the wounded eye, when the other was closed, as he could with the perfect one.

